# In Vivo Imaging of HIF-Active Tumors by an Oxygen-Dependent Degradation Protein Probe with an Interchangeable Labeling System

**DOI:** 10.1371/journal.pone.0015736

**Published:** 2010-12-23

**Authors:** Takahiro Kuchimaru, Tetsuya Kadonosono, Shotaro Tanaka, Takashi Ushiki, Masahiro Hiraoka, Shinae Kizaka-Kondoh

**Affiliations:** 1 Department of Biomolecular Engineering, Graduate School of Bioscience and Biotechnology, Tokyo Institute of Technology, Yokohama, Japan; 2 Department of Biochemistry, School of Medicine, Tokyo Women's Medical University, Tokyo, Japan; 3 Department of Hematology, Graduate School of Medical and Dental Sciences, Niigata University, Niigata, Japan; 4 Department of Radiation Oncology and Image-Applied Therapy, Graduate School of Medicine, Kyoto University, Kyoto, Japan; Massachusetts General Hospital, United States of America

## Abstract

Hypoxia-inducible factor (HIF) functions as a master transcriptional regulator for adaptation to hypoxia by inducing adaptive changes in gene expression for regulation of proliferation, angiogenesis, apoptosis and energy metabolism. Cancers with high expression of the alpha subunit of HIF (HIFα) are often malignant and treatment-resistant. Therefore, the development of a molecular probe that can detect HIF activity has great potential value for monitoring tumor hypoxia. HIF prolyl hydroxylases (HPHDs) act as oxygen sensors that regulate the fate of HIFα protein through its oxygen-dependent degradation (ODD) domain. We constructed a recombinant protein PTD-ODD-HaloTag (POH) that is under the same ODD regulation as HIFα and contains protein transduction domain (PTD) and an interchangeable labeling system. Administration of near-infrared fluorescently labeled POH (POH-N) to mouse models of cancers allowed successful monitoring of HIF-active regions. Immunohistochemical analysis for intratumoral localization of POH probe revealed its specificity to HIF-active cells. Furthermore, lack of the PTD domain or a point mutation in the ODD domain abrogated the specificity of POH-N to HIF-active cells. Overall results indicate that POH is a practical probe specific to HIF-active cell in cancers and suggest its large potential for imaging and targeting of HIF-related diseases.

## Introduction

Hypoxia-inducible factor (HIF) is critical for cellular adaptation in response to limited oxygen availability and regulate a wide array of genes in response to activation by cellular oxygen-sensing mechanisms. In cancers, hypoxia and genetic alterations in oncogenes and tumor suppressor genes increase HIF activity [1]. Many induced by HIF are critically involved in aspects of cancer biology, including immortalization, maintenance of stem cell pools, cellular differentiation, genetic instability, vascularization, metabolic reprogramming, autocrine growth factor signaling, invasion/metastasis and resistance to treatment [2], [3]. Therefore, early detection of HIF-active regions is important to initiate timely and appropriate treatments for malignant cancers.

HIF is a heterodimer of bHLH-PAS proteins. It consists of an alpha subunit (such as HIF-1α), with suppressed degradation during hypoxic conditions, and ubiquitously expressed beta subunits (such as HIF-1β) [1]. Under well-oxygenated conditions, HIFα is hydroxylated by members of the prolyl hydroxylase domain (PHD) family, which target highly conserved prolyl residues located in the oxygen-dependent degradation (ODD) domain [4], [5]. Trans-4-prolyl-hydroxylation of HIFα enables its recognition by the von Hippel-Lindau tumor suppressor protein (pVHL), a component of ubiquitin ligase complexes [6]. HIFα is then polyubiquitinated and undergoes proteasomal degradation when oxygen is available [6]. Hypoxia suppresses the rate of HIF hydroxylation, allowing HIFα to accumulate.

We previously reported that a fusion protein containing ODD_548–603_ of human HIF-1α is efficiently degraded under normoxic conditions through a pVHL-mediated protein degradation system as HIF-1α [7] and that a fusion protein PTD-ODD_548–603_-procaspase-3 significantly increased caspase-3 activity and selectively induced apoptosis in HIF-active/hypoxic cancer cells *in vitro* and *in vivo* [7]-[12]. The protein transduction domain (PTD) transports the fused proteins into cells and allows them to spread throughout the body by passive diffusion [13], [14].

In the present study, we constructed PTD3-ODD-HaloTag (POH), which is easily labeled with any chemical at a specific site through HaloTag, an interchangeable labeling system [15]. We labeled HaloTag with near-infrared fluorescence (NIRF) dyes and injected the resultant PTD3-ODD-HaloTag-ligand-NIRF dye (POH-N) into model mice with cancers, resulting in successful monitoring HIF-1-active [HIF (+)] regions in the model mice. Overall results demonstrate the specificity of PTD3-ODD fusion proteins to HIF (+) cells and their large potential for imaging and targeting of HIF-related diseases.

## Results

### Construction of a near-infrared fluorescently labeled imaging probe

**Figure 1:**
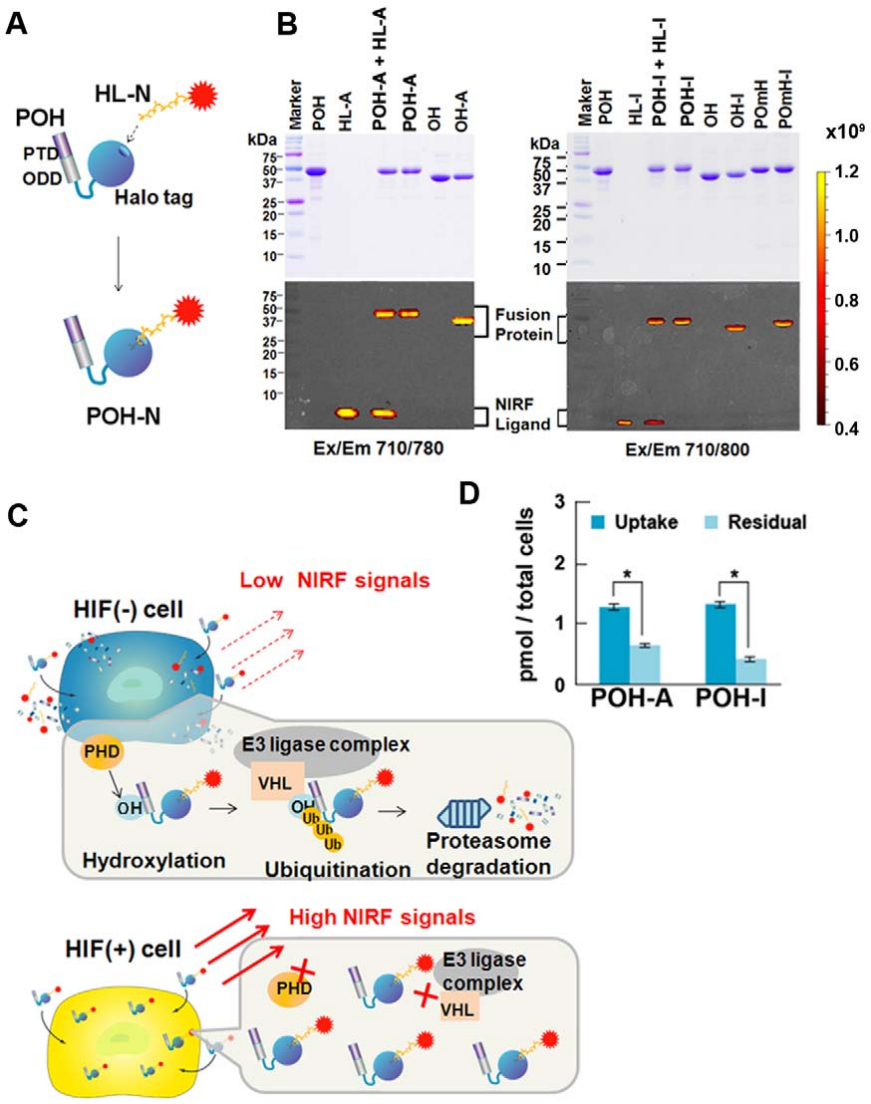
Preparation of POH-N probes. (A) Structure of the POH-N probes. POH fusion protein is composed of PTD, ODD and HaloTag domains. HaloTag ligand labeled with NIRF dye (HL-N) is covalently bound to POH via HaloTag domain to form POH-N probe. (B) SDS-PAGE of prepared probes. PTD-ODD-HaloTag (POH), POmH (POH with a point mutation in the ODD domain) and ODD-HaloTag (OH) fusion proteins were mixed with Alexa Fluor 750- or IR800-labeled HaloTag ligand (HL-A or HL-I) and then unbound HL-A or HL-I was removed by column purification. Probes resolved by SDS-PAGE were stained with Coomassie Blue (upper panel). Fluorescence was scanned by IVIS using excitation (Ex) and emission (Em) filters indicated below the gels (bottom panel). (C) Mechanism of selective imaging for HIF (+) cells. In cells without HIFα (HIF (–) cells), POH-N is immediately degraded through HPHD-mediated ODD and the resultant POH-N fragments and HL-N are diffused from the cells. In cells with HIFα [HIF (+) cells], POH-N is more stable and a clear contrast between cells with versus without HIF activity can be achieved. (D) POH-A and POH-I were added to the culture medium of SUIT2/HRE-Luc cells. After incubation for 1 h under normoxic conditions, the cells were washed and fluorescence intensity was measured (uptake). The cells were further cultured under normoxic conditions for 30 min and fluorescence intensity was measured again (residual). *P<0.01.

The recombinant PTD-ODD-HaloTag (POH) fusion protein was constructed to specifically image and target HIF (+) regions. Although PTD-ODD fusion proteins have been shown *in vivo* to be efficiently delivered to and specifically located in hypoxic regions of the tumors by the function of the PTD and ODD domains, respectively, PTD-ODD fusion proteins with different functions have to be prepared separately. The HaloTag domain endows POH with any function via an interchangeable labeling system, enabling easy preparation of POH with different functions. In this study, POH was fluorescently labeled by the HaloTag interchangeable labeling system and examined for possible application as an *in vivo* imaging probe specific to HIF (+) regions. POH was labeled by covalent binding of near-infrared fluorescent (NIRF) dye-conjugated HaloTag-ligand (HL-N) to a specific site in the HaloTag domain [15] so that the NIRF labeling would not affect the function of the PTD and ODD domains (Figure 1A). For the NIRF dyes, we used two NIRF dyes, IR800 [16] and Alexa Fluor 750. Both have excitation and emission wavelengths in 700–800 nm where non-specific background fluorescence is considerably reduced [17]. The labeling efficiency of POH with HL-IR800 (HL-I) or HL-Alexa Fluor 750 (HL-A) was >0.7 and the final products POH-N [POH-I (PTD-ODD-HL-IR800) and POH-A (PTD-ODD-HL-Alexa Fluor 750)] were stable and did not contain any obvious side products (Figure 1B). The expected process for POH-N to image HIF (+) cells are shown in Figure 1C and required functions for POH-N are as follows: 1) efficient penetration across the cell membrane by the PTD function; 2) rapid degradation of POH by the ODD function in HIF-inactive [HIF (-)] cells; and 3) rapid diffusion of NIRF molecules from HIF(-) cells. We verified the each process with POH-I and POH-A. Rapid diffusion of NIRF molecules from HIF (-) cells was observed with both POH-N probes (Figure 1D).

### PTD in POH-N enables efficient entry into cells and *in vivo* delivery

**Figure 2:**
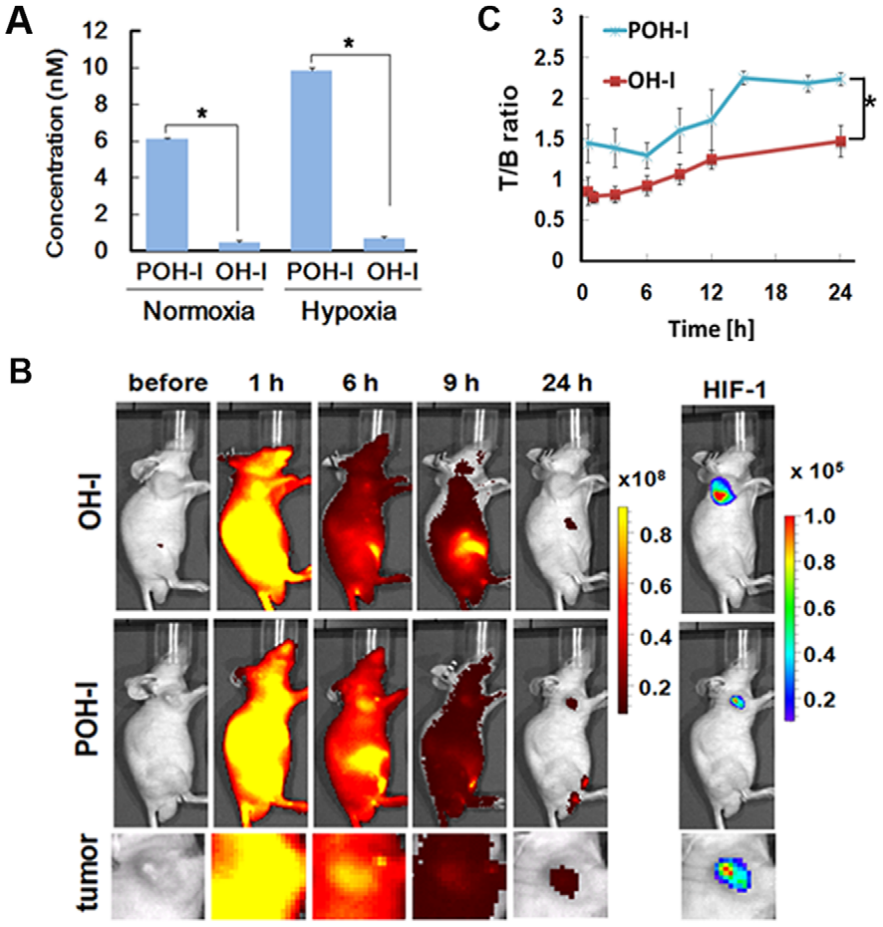
*In vivo* optical imaging of HIF-1 activity in subcutaneous cancers with POH probe and its PTD function *in vitro* and *in vivo.* (A) Membrane permeability of POH-I and OH-I in SUIT-2/HRE-Luc cells. The cells cultured under normoxic or hypoxic conditions were co-cultured with POH-I or OH-I for 1 h and then the fluorescent intensity of cell lysates was measured. The experiments were repeated three times. (B) Representative *in vivo* image after 2 nmol of POH-I or OH-I administration. Nude mice carrying SUIT-2/HRE-Luc xenografts in both forefeet were imaged at the indicated times after probe injection. The right panel (HIF-1) shows bioluminescence images at 24 h. The bottom panels were magnified images of tumor area of middle panels. (C) The relative fluorescence intensity of the tumor to muscle (T/B ratio). Fluorescence intensities of the SUIT-2/HRE-Luc xenografts and the muscle of the hind foot were measured at the indicated times after POH-I administration. *P<0.01, n = 5.

First, efficient penetration across the cell membrane by the PTD function was examined by using POH-I. The POH fusion protein contained PTD3 polypeptide, which has more efficient membrane penetrating activity than known PTD polypeptides in a PTD-ODD fusion protein (Figure S1). To examine the function of PTD3, POH-I without PTD3 (OH-I) was constructed (Figure 1B) and SUIT-2/HRE-Luc cells were treated with OH-I or POH-I. POH-I entered cells much more efficiently than OH-I (Figure 2A), confirming that efficient penetration of the cell membrane is dependent on PTD function [14]. To analyze PTD function *in vivo*, POH-I and OH-I were intravenously injected into mice carrying subcutaneous xenografts of SUIT-2/HRE-Luc cells. Although OH-I and POH-I were delivered to the tumors, the amount and retention time of OH-I in tumors was much less than those of POH-I (Figure 2B). The ratio of the target (fluorescent intensity of tumors) versus background (fluorescent intensity of muscles in the hind legs) (T/B ratio) of POH-I was high (>2.0) 15–24 h after POH-I administration, while the T/B ratio of OH-I was less than 1.5 throughout the observation period (Figure 2C). Similar results were obtained by using POH-A. These results indicate that PTD in POH-N contributes to its efficient entry into tumor cells, and therefore, contributes to the tumor-specific imaging of POH-N.

### The specificity of POH-N to HIF (+) cells is dependent on ODD function

**Figure 3:**
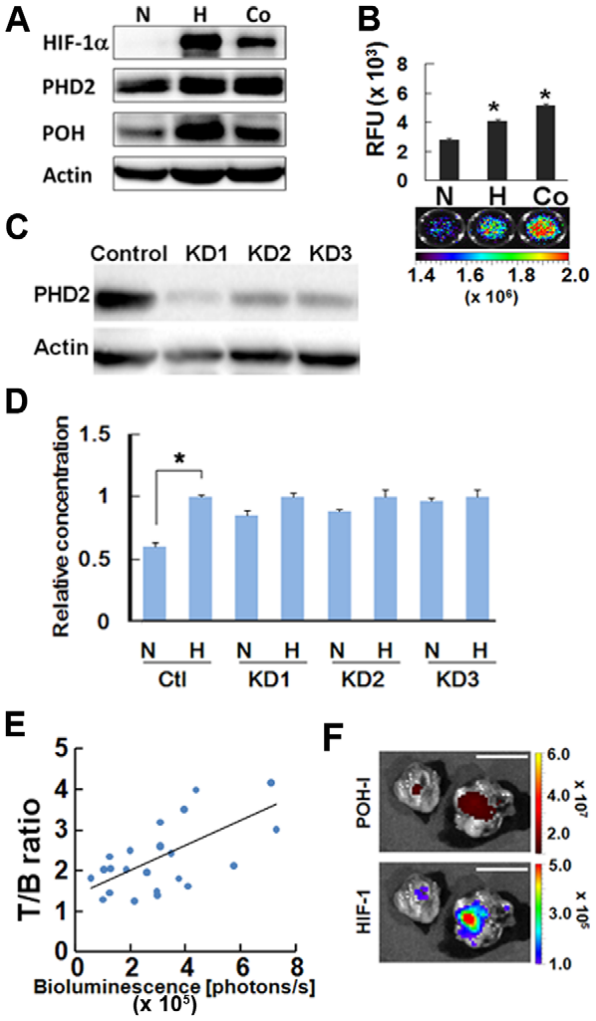
Characterization of POH probe *in vitro* and *in vivo*. (A and B) Correlation of POH stability and the expression of HIF-1α. The SUIT-2/HRE-Luc cells (1.5×10^5^) cultured under normoxic (N) or hypoxic (H) conditions with 250 µM CoCl_2_ (Co), a HPHD inhibitor, were treated with POH protein and analyzed by western blotting (representative blot is shown) (A) and measurement of fluorescent intensity (B) with an Ex/Em wavelength of 710 nm/800 nm (P<0.05, n = 3). Representative fluorescence images are shown below the graphs. (C and D) PHD2 regulation of POH stability. The three independent SUIT-2/HRE-Luc/PHD2-KD cell lines (KD1, KD2 and KD3) with reduced PHD2 protein expression (C), which were isolated after stable transfection of a PHD2-specific shRNA plasmid into SUIT-2/HRE-Luc cells, were cultured under normoxic (N) or hypoxic (H) conditions and were treated with POH-I for 1 h. The cells were cultured in fresh medium and the probes remained in the cells was estimated by the measurement of fluorescent intensity of cell lysates. The experiments were repeated three times and a relative value (hypoxia  = 1) means ± SEM are shown (D). *P<0.05 (vs. normoxic condition). (E) Correlation between tumor bioluminescence intensity and the T/B ratio (relative fluorescence intensity of the tumors to the muscle). Bioluminescence and fluorescent intensities of xenografts were measured 24 h after POH-I administration. Bioluminescence intensity and T/B ratio show the coefficient value of 0.7767. n = 29. (F) Representative *ex vivo* bioluminescence (HIF-1) and fluorescence (POH-I) images of excised subcutaneous tumors. Nude mice carrying SUIT-2/HRE-Luc xenografts in both forefeet were injected with POH-I and dissected 24 h after probe administration. Bar  = 10 mm.

When the cells were treated with POH-I, significantly higher levels of POH protein and NIRF signal were detected in cells cultured in hypoxia (1% O_2_) and in cells treated with CoCl_2_, a PHD inhibitor, compared with normoxic (21% O_2_) cells (Figures 3A and 3B). Similar results were obtained in experiments using other human cancer cell lines (Figure S2). Moreover, when PHD2 expression was significantly suppressed in PHD2-KD subclones by using shRNA (Figure 3C), there were significantly reduced levels of POH-I signal in normoxia than in hypoxia in control SUIT-2/HRE-Luc cells but not in PHD2-KD subclones (Figure 3D). These results indicate that the rapid degradation of POH in HIF (-) cells is regulated by PHD2, which modifies the ODD domain of HIFα under normoxic conditions [18].

When POH-I was injected into mice carrying subcutaneous xenografts of SUIT-2/HRE-Luc cells via the tail vein, fluorescence signals were detected throughout the body of the mice within a few minutes and gradually became weaker (Figure 2B). Accumulation of POH-I in tumors was detected as soon as 6 h after injection (Figure 2B). SUIT-2/HRE-Luc cells stably carry 5HRE-luciferase, a luciferase reporter gene under the control of the HIF-1-dependent promoter, and thus HIF-1-activity in xenografts [HIF (+) regions] can be noninvasively monitored by bioluminescence imaging [12]. Most POH-I was cleared from the mice and tumor-specific images were obtained by 24 h after POH-I administration, resulting in a high T/B ratio (>2.0) in most of the tumors (Figure 3E). A good correlation (R = 0.7767) was observed between bioluminescence intensity of the tumors and the T/B ratio of the POH-I probe (Figure 3E). *Ex vivo* imaging of tumors at 24 h after POH-I injection revealed that most fluorescent images of POH-I overlapped with the bioluminescence images, which indicated HIF (+) regions (Figure 3F). A relatively high level of fluorescence was also observed in some clearance-related organs (Figure S3).

### A point mutation in the ODD domain abrogates the specificity of POH to HIF (+) cells

**Figure 4:**
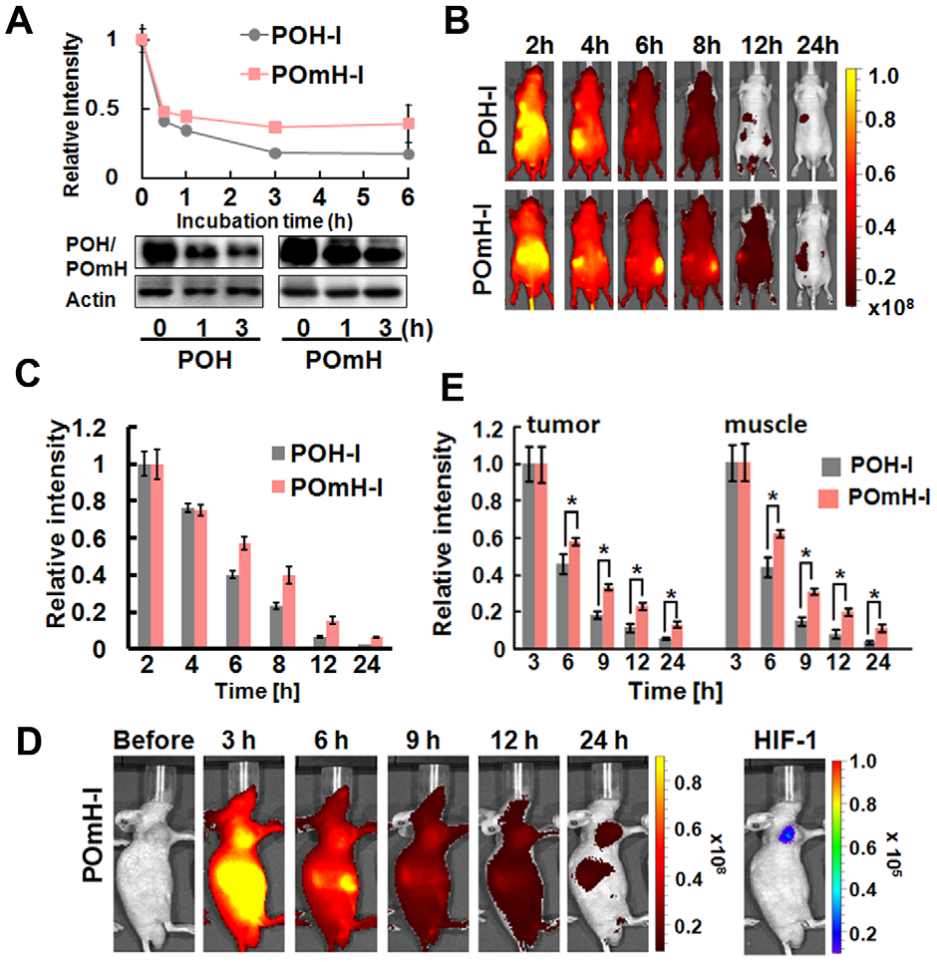
ODD function of POH probe *in vitro* and *in vivo*. (A) Time-dependent degradation of POH-I and POmH-I in HIF (-) cells. SUIT-2/HRE-Luc cells cultured under normoxic conditions were co-cultured with POH-I or POmH-I for 1 h. The cells were cultured in fresh medium and the probes remained in the cells was estimated by the measurement of fluorescent intensity of cell lysates at the represented time-points, shown by the relative value (0 h = 1). Degradation of POH and POmH protein was also detected by western blotting (lower panels). (B and C) Biodistribution of POH-I and POmH-I in the whole body. Mice without xenografting were imaged at the indicated time after 2 nmol intravenous administration of POH-I or POmH-I (B). Fluorescent intensity of the whole mouse was measured at the indicated time after administration of POH-I or POmH-I and relative fluorescent intensities are shown (C). *P<0.01 (n = 4). (D) *In vivo* imaging with POmH-I. Nude mice carrying SUIT-2/HRE-Luc xenografts in both forefeet were injected with 2 nmol of POmH-I and images were taken at the indicated times. The right panel (HIF-1) shows bioluminescence images at 24 h. (E) Fluorescence intensities of tumors and the muscle after POH-I (n = 4) or POmH-I (n = 6) administration were measured at the indicated time and relative fluorescent intensities are shown. *P<0.01.

The contribution of the ODD domain in POH to its HIF (+) cell-specificity was further examined *in vitro* and *in vivo* using POmH-I, which had a point mutation corresponding to human HIF-1α (P564G) in the ODD domain and thus lacked ODD regulation [19]. The relative fluorescent intensity and fusion protein retained in cells treated with POmH-I were significantly higher than in cells treated with POH-I under normoxic conditions (Figure 4A), indicating that ODD contributed to the rapid diffusion of fluorescence from HIF (-) cells. When POH-I and POmH-I were injected into mice via the tail vein, the clearance of POH-I was significantly faster than that of POmH-I in mice without any tumors (Figures 4B and 4C), suggesting that ODD of POH accelerates its clearance from normal tissues. When POmH-I was injected into tumor-bearing mice, tumors were imaged (Figure 4D) and the clearance of POmH-I was significantly slower than that of POH-I. A greater amount of POmH-I was retained in the muscles as well as in the tumors than POH-I throughout the observation period (Figure 4E). These data indicate that ODD contributes to the rapid clearance of POH-I from HIF (-) cells.

### Immunohistochemical analysis reveals the specificity of POH probe to HIF (+) cells in tumors

**Figure 5:**
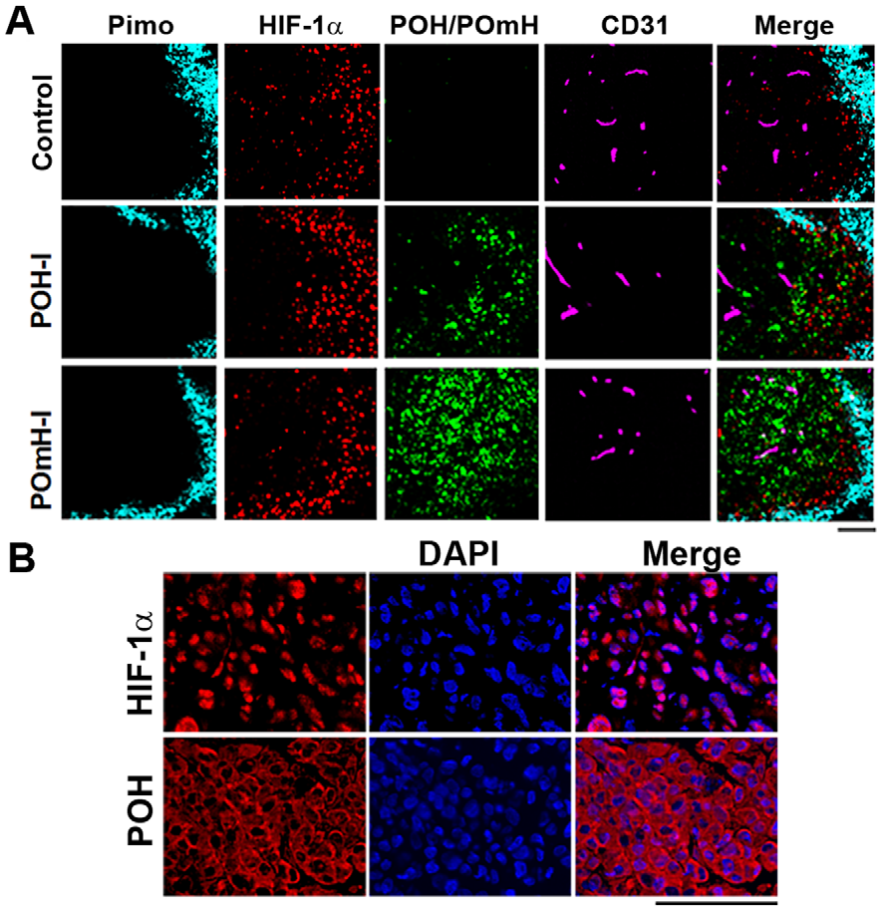
Intratumoral localization of POH probe. (A) Immunohistochemical analyses of SUIT-2/HRE-Luc xenografts. Tumors were removed 6 h after POH-I or POmH-I administration and examined for sever hypoxic regions (Pimo), HIF (+) cells (HIF-1α), probes (POH/POmH) and blood vessel (CD31). Bars  = 100 µm. (B) Cellular localization of HIF-1α and POH. Tumors were excised 6 h after probe injection and examined for cell nuclei (DAPI) and HIF-1α or POH. Bars  = 100 µm.

To examine the specificity of the POH probe to HIF (+) cells, we examined the intratumoral localization of POH in more detail. Tumors were excised 6 h after POH-I or POmH-I injection when the fluorescent signals had significantly decreased throughout the body of the mice (Figure 2B). In tumor sections, pimonidazole-positive severe hypoxic (pO_2_ <10 mmHg) [20] regions were located adjacent to the necrotic regions and generally did not overlap with HIF-1α, as previously described [21]. Both HIF-1α and POH were detected in regions closer to blood vessels than regions positive for pimonidazole, and mostly overlapped (Figure 5A). Conversely, POmH was widely distributed around the blood vessels (Figure 5A). These results confirmed that POH was efficiently delivered to and was located in HIF (+) regions in tumors. In tumor cells, HIF-1α was located in cell nuclei, while POH was predominantly located in the cytoplasm (Figure 5B), consistent with the fact that POH lacks the C-terminal nuclear localization signal of HIFα, which is important for HIF nuclear translocation [22]. Overall, these results demonstrate the specificity of POH to HIF (+) cells.

### *In vivo* imaging of HIF-1-active cancer cells in an orthotopic pancreatic cancer model

**Figure 6:**
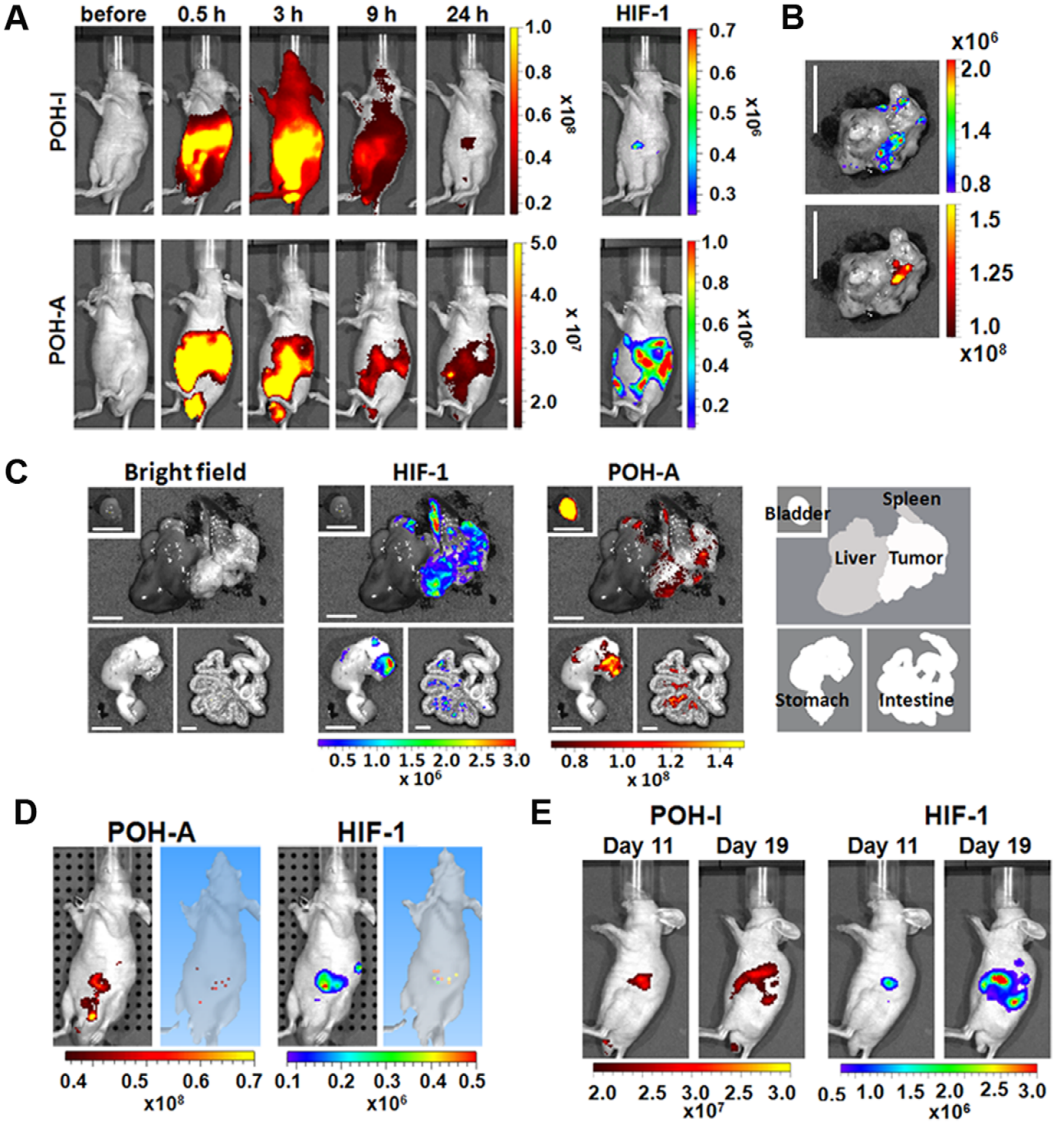
*In vivo* optical imaging of HIF-1 activity in an orthotopic pancreatic cancer model with POH probes. (A) *In vivo* imaging of pancreatic cancer. Nude mice were orthotopically injected with SUIT-2/HRE-Luc cells and 11 days (upper panels) or 19 days (bottom panels) later the mice were intraperitoneally injected with POH-I or POH-A and imaged at the indicated times after probe administration. Bioluminescence images (HIF-1) of the mice 24 h after probe administration are shown in the right panels. (B and C) *Ex vivo* imaging of pancreatic cancer. The mice shown in the upper and lower panels of (A) were analyzed by *ex vivo* imaging and shown in (B) and (C), respectively. Fluorescence (POH-I or POH-A) and bioluminescence (HIF-1) images at 24 h are shown. Bar  = 10 mm (B) or 5 mm (C). (D) Three dimensional analysis of acquired fluorescent (POH-A) and bioluminescent (HIF-1) images. A mouse with pancreatic cancer was injected with POH-A 10 days after orthotopic transplantation and were imaged 24 h after POH-A injection. (E) A mouse with pancreatic cancer was injected with POH-I 11 and 19 days after orthotopic transplantation. Fluorescence (POH-I) and bioluminescence (HIF-1) images were taken 24 h after POH-I injection on the indicated days.

Next, we examined if POH-N was able to detect small localized and metastasized tumors in an orthotopic pancreatic cancer model, in which HIF (+) cancer cells play a central role in pancreatic cancers during local invasion and metastasis [12]. Fluorescent images corresponding to the images of bioluminescent signals, indicating HIF (+) cancer cells, were obtained at 9–24 h after intraperitoneal injection of POH-I (Figure 6A) and successfully detected a small tumor localized in the pancreas (Figure 6B). Small metastasized pancreatic cancers in the peritoneum and stomach were also detected by POH-A (Figure 6C). We also evaluated intravenous injection of POH-N, but this proved unsuccessful because the tumor was not effectively labeled (Figure S4). No fluorescent signal was detected in the abdominal organs of mice or HIF (+) cancer with bioluminescent signals (Figure S5), indicating that neither autofluorescence nor bioluminescence influenced specific image acquisition. Fluorescent 3D images corresponding to 3D images of bioluminescent signals further confirmed the specificity of the POH probe to the HIF (+) pancreatic cancers (Figure 6D). We also successfully monitored the progression of pancreatic cancer by repeated injections of POH-I in the same mice (Figure 6E). Taken together, these results indicate that POH is a practical probe to detect HIF (+) cancers in orthotopic cancer models as well as subcutaneous cancer models.

## Discussion

We report, for the first time to our knowledge, non-invasive fluorescent monitoring of HIF-active microenvironments in cancer in living animals using a oxygen-dependent degradation protein probe, POH. Many hypoxia-specific probes have been reported, including positron emission tomography (PET) probes such as ^18^F-FMISO [23], ^18^F-FAZA [24] and ^64^Cu-ATSM [25]. These label hypoxic regions with reductase activity and have been used to image hypoxic tissues in tumors, mice and humans. In contrast, POH is more specific for HIF (+) regions, which are closely associated with the relatively mild hypoxic status of cells and do not overlap with regions labeled by pimonidazole (Figure 5 and ref. [21]), a nitroimidazole compound similar to FMISO, that targets reductases to detect severely hypoxic regions (pO_2_≤10 mmHg) [20]. Furthermore, in cancers, HIF activity does not always reflect intratumoral hypoxic status. HIFα expression is increased by genetic changes in the components of the PI3K-mTOR growth signal pathways, in oncogenes products such as ras and raf or in the von Hippel-Lindau and p53 tumor suppressor proteins [1], [3], [26]–[29]. Therefore, there is an urgent need for an imaging probe to detecting HIF (+) cells. Many luciferase reporter genes, which specifically image HIF (+) cells, have been developed [30]–[32]. However, the use of luciferase reporter genes is limited because endogenous expression of the reporter genes in target cells is required. The current study is the first to demonstrate an exogenous optical probe specific to HIF (+) cells. We present three prerequisites for an optical HIF (+)-specific exogenous protein probe (Figure 1C) and demonstrate that POH-N fits these criteria (Figures 1, 2, 3, 4, 5).

Whole-body imaging using bioluminescence and fluorescence can greatly refine our understanding of small animal models of human physiology and disease [17], [33], [34]. We took advantage of the multiple optical imaging techniques and evaluated the target specificity of fluorescently labeled POH by using HIF-specific bioluminescence imaging in cancer mouse models (Figures 2, 3, 4, 6).

We failed to detect pancreatic cancer after intravenous injection of POH-N probes (Figure S4), possibly due to preferential uptake of the probe by the liver before reaching the pancreas. However, it is also possible that the pancreas showed poor uptake of the probes or less blood flow to the xenografts.

In our experiments, OH-I was delivered to the tumors in detectable amounts and imaged tumors (Figure 2B) probably due to high blood supply to tumors [35]. However, its retention time was significantly shorter than POH-I and it did not give clear images of the tumors (Figure 2B), indicating that PTD increases the retention of POH-I by transducing it into tumor cells. POH-I was also delivered to regions with less blood flow, including the hypoxic regions of tumors (Figure 5A), most probably by diffusion. These results further support the idea that PTD allows PTD-ODD fusion proteins to be delivered to regions far from blood vessels in tumors and support previous studies in which hypoxic/HIF (+) cancer cells were eradicated by PTD-ODD-procaspase-3 [7]–[12].

POH-N was successfully detect ischemic lesions in mouse models for ischemic diseases such as focal cerebral ischemia (Fujita et al. in preparation) and myocardial infarction (Watanabe et al. in preparation), suggesting that POH could be used to deliver any drug selectively to hypoxic tissue, including the penumbra, which has potential for recovery, and therefore, is a target for medical interventions.

POH was significantly accumulated in tumors (Figure 2B), and had been specifically detected in HIF (+) cells (Figure 5) 3–6 h after administration, although some strong fluorescent signals were also detected in clearance organs, especially in the bladder; this is also observed in clinical PET imaging such as ^18^F-Fluorodeoxyglucose-PET. Currently, clinical application of optical imaging probes is limited to endoscopic and intraoperative uses, and thus clinical application of POH-N would be limited. However, POH-N would be a useful tool for translational studies using small animals. Moreover, as the HaloTag ligand can be conjugated to a wide range of biomaterials, including radioisotopes and anti-apoptotic agents through an interchangeable labeling system, POH offers sensitive imaging and targeting bioprobes with specificity for HIF (+) cells; POH can be used in a wide range of applications, including as imaging probes for PET, single photon emission computed tomography and MRI, to deliver drugs for treatment of cancer and ischemic diseases, and to analyze the biology of HIF. Further studies are needed to evaluate the tissue specificity and pharmacokinetic characteristics of POH to determine its most appropriate clinical uses, as well as identify the most appropriate sites of administration to avoid unwanted accumulation or rapid excretion, based on the target tissue.

## Materials and Methods

### Ethics statement

All animal experiments were performed with the approval of the Animal Experiment Committees of Kyoto University Graduate School of Medicine and conducted according to relevant national and international guidelines.

### Mice

Male Balb/c nu/nu were purchased from Oriental Yeast Co., Ltd. All mice underwent experiments at 6–10 weeks of age.

### Cells and culture conditions

In SUIT-2/HRE-Luc cells, HIF-1α stability is strictly regulated by oxygen concentration (HIF-1α expression is undetectable under normoxic conditions but significantly increased under hypoxic conditions [12]. SUIT2/HRE-Luc/PHD2-KD cells were cloned after transfection with a pRS shRNA vector encoding a 29 mer shRNA specific for PHD2 (Origene, Rockville, MD, USA) into SUIT2/HRE-Luc cells. Luciferase-expressing subclones under normoxic conditions were selected. All the cells were maintained at 37°C in 5% FCS-Dulbecco's-modified Eagle's medium (Nacalai Tesque, Kyoto, Japan) supplemented with penicillin (100 units/ml) and streptomycin (100 µg/ml). Hypoxic cell cultures were performed in 5% CO_2_/1% O_2_ in a multi-gas incubator (ASTEC, Fukuoka, Japan).

### Plasmid Construction

The plasmid encoding PTD-ODD-HaloTag7 protein (POH) was constructed by substituting coding sequences of procaspase-3 in the PTD3-ODD-procaspase-3 (POP33)[12] to HaloTag7 (Promega, Madison, WI, USA). The plasmid encoding POmH, which contained the point substitution mutation (P→G)[19] at the proline residue corresponding to HIF-1α P564 was prepared by using the QuickChange XL site-directed mutagenesis kit (Stratagene, La Jolla, CA, USA). Final cDNA constructs were inserted into the plasmid pGEX-6P-3 (GE Healthcare Bio-Science Corp., Piscataway, NJ, USA).

### Preparation of fusion proteins

Fusion proteins were expressed in BL21-CodonPlus cells (Stratagene, La Jolla, CA, USA) as GST-tagged proteins. The GST-tagged proteins were a prepared essentially as previously described [12], purified with a GST-column and digested with precision protease (GE healthcare Bio-Science Corp.) to remove GST-tag from the fusion proteins. The final products were equilibrated in Mg^2+^-/Ca^2+^-free PBS (pH 8.0).

### Isolation of PHD2-KD clones

The PHD2-KD cell lines were obtained by stable transfection of a PHD2-specific shRNA plasmid into SUIT-2/HRE-Luc cells and the KD1, KD2 and KD3 clones were selected by the indication of high luciferase expression under normoxic conditions.

### Preparation of the POH-I and POH-A probes

The HaloTag ligand-IR800 was provided by Promega Corporation (Madison, WI, USA). HaloTag Amine (O4) Ligands (1 mg, 2.87 µmol, Promega) in 100 µl of dimethyl formamide (DMF) were mixed with Alexa Fluor 750 succinimidyl ester (1 mg, ∼0.8 µmol, Invitrogen, Carlsbad, CA, USA) in 1 ml of 10 mM boric acid (pH 8.5) in DMF. The reaction mixture was stirred for 12 h at room temperature in the dark. The reaction mixture was applied to a SepPak C18 reverse-phase column (Waters, Milford, MA, USA) and the HaloTag Ligands labeled with near-infrared fluorescence substances (HL-N) were resolved in 100 µl of DMF. POH protein (40 µmol/L) was mixed with 2 volumes of HL-N (80 nmol/15 µl) in 10 ml of PBS (pH 8.0) containing 100 mM Tris-HCl (pH 8.0) and 3 M (NH_4_)_2_SO_4_ for 2 h. Then, the POH-N probes were purified with a PD-10 gel filtration column (GE Healthcare, Waukesha, WI, USA) and an Amicon-10 centrifugation column (Millipore, Milford, MA, USA). The purified POH-N was finally resolved in PBS (pH 8.0). Fluorescence characterizations were confirmed by SDS-PAGE fluorescence imaging and the labeling rate was calculated as described by the manufacturer. The labeling rate was >0.7.

### Preparation of the polyclonal anti-ODD antibody

The polyclonal anti-ODD antibody was provided by Oriental Yeast Co., Ltd. The polypeptide QDTDLDLEMLAPYIPMDDDFQLRSFDQLSPLESSSA was conjugated to KHL and injected into two rabbits at 4-week intervals. The serum was harvested 8 weeks after the first immunization and purified with an affinity column. The specificity of the purified antibody to PTD-ODD fusion proteins was examined by western blotting and fluorescence microscopic observation (Figure S6).

### Western blotting

For analysis of cultured cells, SUIT-2/5HRE-Luc cells (2.0×10^5^ cells/well) were seeded in a six-well plate. The cells were pre-incubated under aerobic or hypoxic conditions with or without 250 µM CoCl_2_ for 5 h. Then, 50 µg of the probe was added and incubated for 1 h under the same conditions as for preincubation. The cells then were washed with medium, incubated for 3 h, and lysed using 200 µl of Laemmli sample buffer. The protein samples were electrophoresed on 12.5% (for ODD, PHD2 and actin) or 10% (for HIF-1α) SDS-polyacrylamide gel and transferred to Hybond ECL membranes (GE Healthcare). The POH-N probe, actin and HIF-1α were detected with 10 µg/ml of a polyclonal anti-ODD antibody, 0.75 µg/ml of a monoclonal anti-actin antibody (Sigma Life Science), and 0.25 µg/ml of a monoclonal anti-HIF-1α antibody (BD Biosciences Pharmingen, San Diego, CA, USA), respectively. The primary antibodies were then reacted with appropriate secondary horseradish peroxidase-conjugated antibodies (GE Healthcare). Signals were detected using the chemiluminescent ECL-PLUS system (GE Healthcare).

### Immunohistochemical analysis

Samples were embedded in Tissue-Tek OCT compound (Sakura Finetechnical, Tokyo, Japan) in dry ice/ethanol baths. Cryosections (10 µm thick) were prepared using a cryostat (Leica CM3050S, Leica Microsystems, Wetzlar, Germany) and fixed in 4% paraformaldehyde. Then sections were incubated with blocking solution (1% BSA, Nacalai Tesque) for 30 min at room temperature. The primary antibodies were incubated overnight at 4°C. TRITC-conjugated swine anti-rabbit (1/40 dilution in PBS, Dako, Glostrup, Denmark) and Alexa Fluor 680-labeled goat anti-rat (1/50 dilution in PBS, Invitrogen) secondary antibodies, as appropriate, were used to fluorescently label the corresponding primary antibodies. Cryosections (10 µm thick) were incubated for 1 h with FITC-conjugated Hypoxyprobe-1 primary antibody supplied with the kit (Hypoxyprobe-1 Plus Kit, Chemicon International). For DAPI staining, the sections were mounted in Vectashield with DAPI (Vector Laboratories, Burlingame, CA, USA). All photos were taken using a BZ-9000 microscope (Keyence, Osaka, Japan).

### Transplantation of subcutaneous and orthotopic pancreatic cancer xenografts

For subcutaneous xenografts, SUIT-2/HRE-Luc cells suspended in PBS (1.0×10^6^ cells/20 µl) were mixed with an equal volume of Geltrex (Invitrogen) and injected into both forelegs of 7-week-old male nude mice. For the orthotopic pancreatic cancer model, SUIT-2/HRE-Luc cells (2×10^5^ cells/50 µl) were mixed in an equal volume of Geltrex (Invitrogen) and were injected into the pancreas of 8-week-old male nude mice. Mice with subcutaneous tumors of 5–15 mm in diameter were used for experiments.

### *In vivo* and *ex vivo* bioluminescence imaging

For *in vivo* photon counting to assess bioluminescence, tumor-bearing mice were intraperitoneally injected with 200 µl of D-luciferin solution (10 mg/ml in PBS, Promega) and placed in an IVIS®-Spectrum *in vivo* photon-counting device (Caliper Life Sciences, Alameda, CA, USA). Bioluminescence images were acquired at 20 min for subcutaneous xenografts and 13 min for the pancreatic cancer model after the intraperitoneal injection of D-luciferin. For *ex vivo* imaging, randomly selected mice were sacrificed immediately after the *in vivo* imaging and bioluminescence images of the bodies and their organs were measured within 10 min after *in vivo* imaging. The following conditions were used for image acquisition: exposure time  = 2 min (for *in vivo*) or 1 min (for *ex vivo*), lamp level  =  high, binning  =  medium:8, field of view  = 19×19 cm (for *in vivo*), 12.9×12.9 cm (for *ex vivo*), and f/stop  = 1. The bioluminescence was analyzed by Living Image 3.10 software (Caliper Life Sciences). Because the background bioluminescence of untreated nude mice (with D-luciferin injection) was less than 4000 photon counts/s, we considered signals of >1×10^4^ photon counts/s as significant. The minimum and maximum photons/s/cm^2^/sr of each image are indicated in each figure by a rainbow bar scale.

### *In vitro* fluorescent imaging

SUIT-2/HRE-Luc, A549 lung adenocarcinoma, or HeLa human cervical cancer cells (2×10^5^ cells/well) were seeded in a six-well plate. The cells were pre-incubated under aerobic or hypoxic conditions for 16 h with or without 250 µM CoCl_2_ for 5 h, followed by the addition of 500 nM of probe, and incubated for 1 h. The cells were then washed with fresh medium, incubated for indicated time in fresh medium, and suspended in 200 µl of radio-immunoprecipitation assay (RIPA) buffer. Fluorescent imaging was performed for 150 µl of the suspensions in the 96-well plate.

### *In vivo* fluorescence imaging

Two nmol of POH-N, OH-I and POmH-I in 100 µl of PBS (pH 8.0) were injected into tumor-bearing mice intravenously or intraperitoneally. Fluorescence images were acquired at the indicated times. All of the fluorescence images were acquired with IVIS®- Spectrum (Caliper Life Sciences) using the same parameters for the excitation filter (710±15 nm), emission filter (780±10 nm for POH-A; 800±10 nm for POH-I, OH-I and POmH-I) and exposure time  = 1 s, binning  =  medium (8), field of view  = 19×19 cm, f/stop = 1.

### Analysis of T/B ratio

Photon flux/sec in the same area (region of interest: ROI) of the tumors and the muscle in the lower limbs were counted at the indicated time after probe injection. Each photon flux/sec was normalized by subtracting photon flux/sec in the corresponding ROI of an untreated mouse. T/B was calculated by dividing the normalized values in tumors by the ones in the muscle.

### *Ex vivo* optical imaging

Randomly selected mice were sacrificed after the *in vivo* imaging and organs were harvested. Fluorescence emissions from these organs were measured with the IVIS Spectrum system using the same parameters for the excitation filter (710±15 nm), emission filter (780±10 nm for POH-A; 800±10 nm for POH-I and OH-I and POmH-I), and exposure time (1 s). In addition, bioluminescence images were acquired to assess HIF-1 activity in tumors with a 1-min exposure time. The additional following conditions: lamp Level  =  high, binning  =  medium (8), field of view  = 12.9×12.9 cm, f/stop = 1 were used for image acquisition.

### Three-dimensional fluorescence imaging

Surface topography and transillumination imaging system were used to obtain information for constructing a three-dimensional fluorescence image of 24 h after POH-A administration in an orthotopic pancreatic cancer mouse and 6 h after POH-I administration in a brain ischemic mouse. A series of fluorescence images were taken at 17 different transillumination points around the abdominal area for the pancreatic cancer model. The following conditions: exposure time  = 1 s, binning  =  medium (8), field of view  = 19×19 cm, f/stop  = 1 and excitation (710±15 nm) and emission (780±10 nm) filters were used for image acquisition. For three-dimensional bioluminescence imaging in the pancreatic cancer model, bioluminescence images were acquired with three different wavelength (600±10, 620±10, 640±10 nm) of bioluminescence filters. The following conditions were used for image acquisition: exposure time  = 2 min, lamp level  =  high, binning  =  medium (8), field of view  = 19×19 cm and f/stop  = 1.

### Analysis of cell penetrating activity of PTD-ODD-EGFP fusion proteins by fluorescence-activated cell sorter

Expression vector plasmids for the PTD fusion proteins were constructed by inserting the cDNA fragments encoding PTD, which was prepared by annealing the corresponding oligonucleotides, ODD and EGFP, which were prepared by PCR amplification with the corresponding primers, into the plasmid pGEX-6P-3. Recombinant proteins were prepared as previously described [12], suspended in PBS at a final concentration of 50 µM and added to the culture medium of Suit-2 cells at the final concentration of 50 µg/ml. The cells were incubated at 37°C for 30 min, trypsinized and suspended in 300 µL of icecold PBS. The fluorescence intensity of the cells was determined by fluorescence-activated cell sorting using CellQuest (Becton Dickinson).

### Statistical analysis

Statistical analyses were carried out with a Student's t test. Values of *P*<0.05 were considered statistically significant. Pearson's correlation coefficient was also calculated for evaluation of the correlation of data sets.

## Supporting Information

Figure S1 **The cell penetrating activity of PTD-ODD fusion proteins.** (A) Evaluation of PTD membrane permeability. Left panel shows a representative FACS analysis of EGFP, which did not penetrate the cell membrane and was used as a negative control (NC) and nona-Lys-ODD-EGFP (K9OE). The peaks corresponding to the fluorescence intensity (y) of PTD-ODD-EGFP proteins were divided by the peak of fluorescence intensity of EGFP (x) and are indicated on the graph as relative fluorescence intensity (right panel). The experiments were done in triplicate and repeated three times. Results are indicated as mean ± SEM. P3OE: PTD3-ODD-EGFP, K9OE: 9K-ODD-EGFP, R9OE: 9R-ODD-EGFP, TAT-OE: Tat-ODD-EGFP.
(TIF)

Figure S2 **The specificity of POH-I probe to HIF (+) cells** ***in vitro.*** A549 (a human lung adenocarcinoma cell line) and HeLa (a human cervical cancer cell line) cells cultured under normoxic (N) or hypoxic (H) conditions or normoxic conditions in the presence of 250 µM CoCl_2_ (Co) were treated with POH-I. Fluorescent intensity of cell lysates of POH-I treated cells were measured at an Ex/Em wavelength of 710 nm/800 nm. The experiments were repeated three times and the results are presented as mean ± SEM. *P<0.05 vs. the normoxic condition. Representative fluorescence images taken by IVIS are shown below the graph.
(TIF)

Figure S3 **Representative** ***ex vivo*** **florescent images of organs 24 h after POH-I injection.** Mice with subcutaneous xenografts of SUIT-2/5HRE-Luc cells were injected intravenously with 2 nmol of POH-I. Representative *ex vivo* images of organs at 24 h after POH-I injection are shown. 1-heart, 2-lung, 3-kidney, 4-liver, 5-muscle, 6-spleen, 7-stomach, 8-intestine.
(TIF)

Figure S4 ***In vivo*** **optical imaging of orthotopic pancreatic cancer mice after intravenous injection with the POH probe.** Mice were injected with 1 nmol of POH-A via the tail vein. The fluorescence images were taken at the indicated times after POH-A injection. Bioluminescence imaging (HIF-1) was taken 24 h after POH-A injection.
(TIF)

Figure S5 **Representative** ***ex vivo*** **optical images of organs from an orthotopic pancreatic cancer model mouse without POH probe injection.** Tumor-bearing mice were sacrificed on day 19 after transplantation and bright field, bioluminescence and fluorescence images were taken. Representative images for one mouse are shown. No autofluorescence was observed with the imaging settings used (Ex/Em = 710/780 nm). The right panel indicates the labels of the organs depicted in the other panels (1: muscle; 2: heart; 3: liver; 4: lung; 5: kidneys; 6: bladder; 7: testis; 8: intestine; 9: stomach; 10: pancreatic tumor; 11: seminal vesicles; 12: testicle).
(TIF)

Figure S6 **Evaluation of the polyclonal anti-ODD antibody for western blotting and immunohistochemical analysis.** (A) Western blotting was performed to evaluate the specificity of the anti-ODD antibody. The indicated amounts of purified POH protein and total cell lysate (Cell Lys) of SUIT-2/HRE-Luc cells cultured under normoxic (N) or hypoxic (H) conditions were electrophoresed on 12.5% (left) or 10% (right) SDS-polyacrylamide gels and transferred to nitrocellulose membranes. The resultant membranes were probed with 1 µg/ml of the anti-ODD antibody (left) or 250 ng/ml of the monoclonal anti-HIF-1α antibody (right). Cross-reactivity of the polyclonal anti-ODD antibody to HIF-1α was negligible. The expected molecular weights of HIF-1α and POH are indicated by arrowheads. (B) Cellular immunostaining was examined for the POH-treated and PBS-treated (control) SUIT-2/HRE-Luc cells with anti-ODD antibody (green) and DAPI (blue). Cells (5×10^4^) were cultured in a slide chamber and incubated with 10 µg of POH for 30 min, washed with PBS and fixed in 4% paraformaldehyde. The fixed cells were immunostained with 100 µg/ml of anti-ODD antibody. DAPI staining was also performed according to the manufacturer's instructions. Bar  = 100 µm. Magnified images of POH-treated cells are shown in the lower left corner. Bar  = 10 µm.
(TIF)
