# Supplementary figures and images for: In Vivo Imaging of HIF-Active Tumors by an Oxygen-Dependent Degradation Protein Probe with an Interchangeable Labeling System
Source: PLoS One. 2010 Dec 23;5(12):e15736. doi: 10.1371/journal.pone.0015736 (PMC3009742; doi:10.1371/journal.pone.0015736)

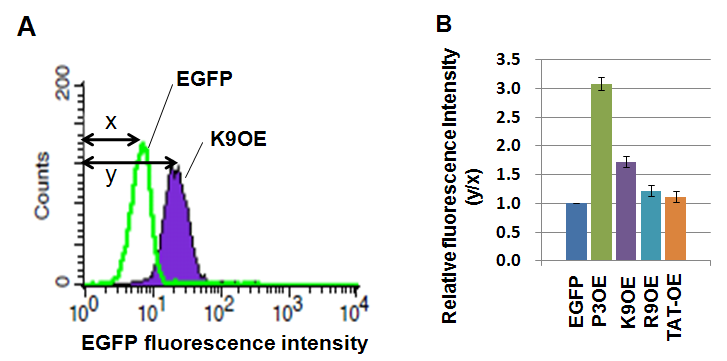

Supplement: Figure S1 — The cell penetrating activity of PTD-ODD fusion proteins. (A) Evaluation of PTD membrane permeability. Left panel shows a representative FACS analysis of EGFP, which did not penetrate the cell membrane and was used as a negative control (NC) and nona-Lys-ODD-EGFP (K9OE). The peaks corresponding to the fluorescence intensity (y) of PTD-ODD-EGFP proteins were divided by the peak of fluorescence intensity of EGFP (x) and are indicated on the graph as relative fluorescence intensity (right panel). The experiments were done in triplicate and repeated three times. Results are indicated as mean ± SEM. P3OE: PTD3-ODD-EGFP, K9OE: 9K-ODD-EGFP, R9OE: 9R-ODD-EGFP, TAT-OE: Tat-ODD-EGFP. (TIF) [file pone.0015736.s001.tif]

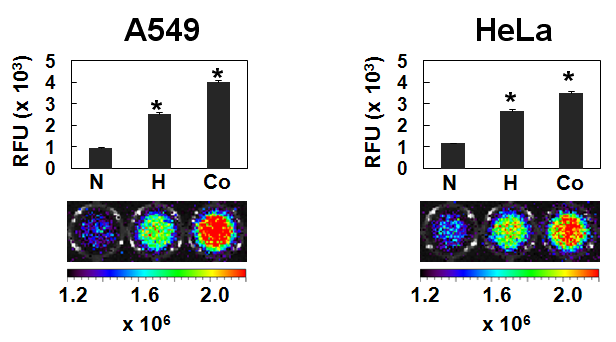

Supplement: Figure S2 — The specificity of POH-I probe to HIF (+) cells in vitro. A549 (a human lung adenocarcinoma cell line) and HeLa (a human cervical cancer cell line) cells cultured under normoxic (N) or hypoxic (H) conditions or normoxic conditions in the presence of 250 µM CoCl2 (Co) were treated with POH-I. Fluorescent intensity of cell lysates of POH-I treated cells were measured at an Ex/Em wavelength of 710 nm/800 nm. The experiments were repeated three times and the results are presented as mean ± SEM. *P<0.05 vs. the normoxic condition. Representative fluorescence images taken by IVIS are shown below the graph. (TIF) [file pone.0015736.s002.tif]

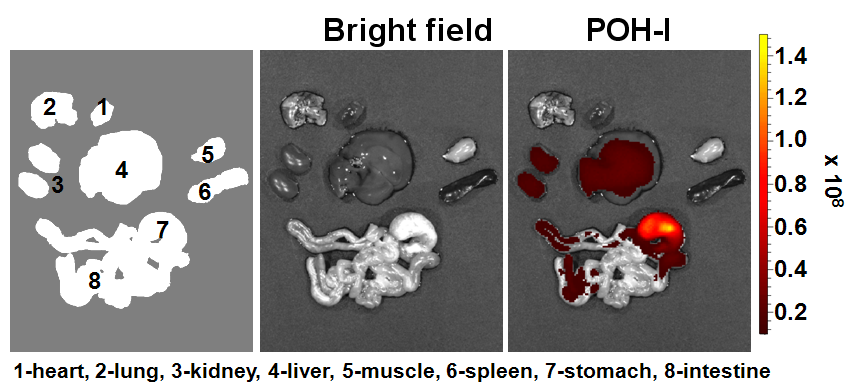

Supplement: Figure S3 — Representative ex vivo florescent images of organs 24 h after POH-I injection. Mice with subcutaneous xenografts of SUIT-2/5HRE-Luc cells were injected intravenously with 2 nmol of POH-I. Representative ex vivo images of organs at 24 h after POH-I injection are shown. 1-heart, 2-lung, 3-kidney, 4-liver, 5-muscle, 6-spleen, 7-stomach, 8-intestine. (TIF) [file pone.0015736.s003.tif]

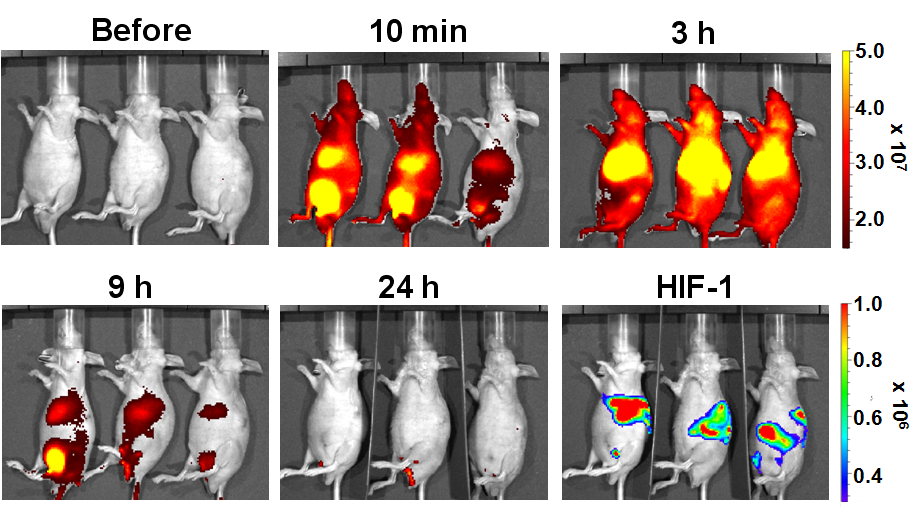

Supplement: Figure S4 — In vivo optical imaging of orthotopic pancreatic cancer mice after intravenous injection with the POH probe. Mice were injected with 1 nmol of POH-A via the tail vein. The fluorescence images were taken at the indicated times after POH-A injection. Bioluminescence imaging (HIF-1) was taken 24 h after POH-A injection. (TIF) [file pone.0015736.s004.tif]

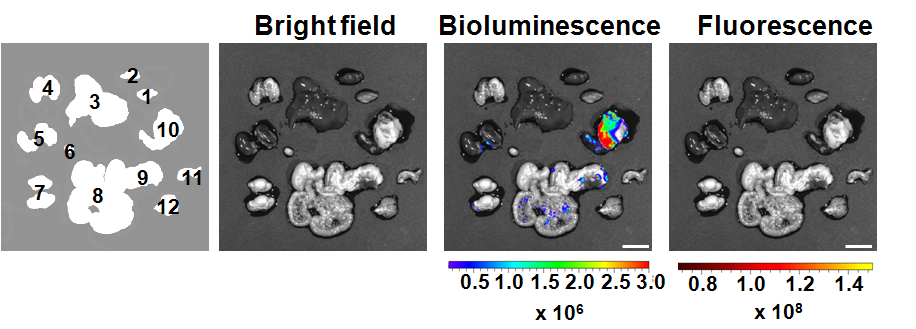

Supplement: Figure S5 — Representative ex vivo optical images of organs from an orthotopic pancreatic cancer model mouse without POH probe injection. Tumor-bearing mice were sacrificed on day 19 after transplantation and bright field, bioluminescence and fluorescence images were taken. Representative images for one mouse are shown. No autofluorescence was observed with the imaging settings used (Ex/Em = 710/780 nm). The right panel indicates the labels of the organs depicted in the other panels (1: muscle; 2: heart; 3: liver; 4: lung; 5: kidneys; 6: bladder; 7: testis; 8: intestine; 9: stomach; 10: pancreatic tumor; 11: seminal vesicles; 12: testicle). (TIF) [file pone.0015736.s005.tif]

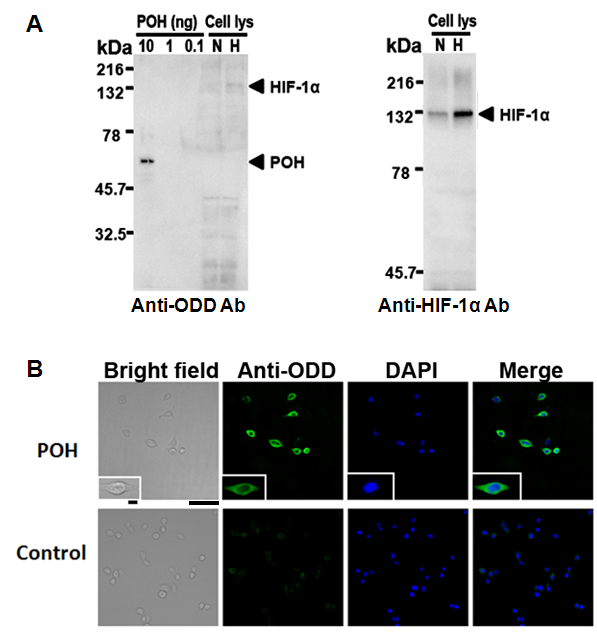

Supplement: Figure S6 — Evaluation of the polyclonal anti-ODD antibody for western blotting and immunohistochemical analysis. (A) Western blotting was performed to evaluate the specificity of the anti-ODD antibody. The indicated amounts of purified POH protein and total cell lysate (Cell Lys) of SUIT-2/HRE-Luc cells cultured under normoxic (N) or hypoxic (H) conditions were electrophoresed on 12.5% (left) or 10% (right) SDS-polyacrylamide gels and transferred to nitrocellulose membranes. The resultant membranes were probed with 1 µg/ml of the anti-ODD antibody (left) or 250 ng/ml of the monoclonal anti-HIF-1α antibody (right). Cross-reactivity of the polyclonal anti-ODD antibody to HIF-1α was negligible. The expected molecular weights of HIF-1α and POH are indicated by arrowheads. (B) Cellular immunostaining was examined for the POH-treated and PBS-treated (control) SUIT-2/HRE-Luc cells with anti-ODD antibody (green) and DAPI (blue). Cells (5×10^4^) were cultured in a slide chamber and incubated with 10 µg of POH for 30 min, washed with PBS and fixed in 4% paraformaldehyde. The fixed cells were immunostained with 100 µg/ml of anti-ODD antibody. DAPI staining was also performed according to the manufacturer's instructions. Bar = 100 µm. Magnified images of POH-treated cells are shown in the lower left corner. Bar = 10 µm. (TIF) [file pone.0015736.s006.tif]
